# Cigarette Smoking, Health-Related Behaviors, and Burnout Among Mental Health Professionals in China: A Nationwide Survey

**DOI:** 10.3389/fpsyt.2020.00706

**Published:** 2020-07-17

**Authors:** Lei Xia, Feng Jiang, Jeffrey Rakofsky, Yulong Zhang, Kai Zhang, Tingfang Liu, Yuanli Liu, Huanzhong Liu, Yi-lang Tang

**Affiliations:** ^1^ Department of Psychiatry, Chaohu Hospital of Anhui Medical University, Hefei, China; ^2^ Department of Psychiatry, Anhui Psychiatric Center, Anhui Medical University, Hefei, China; ^3^ Public Health School, Chinese Academy of Medical Sciences and Peking Union Medical College, Beijing, China; ^4^ Research Center for Public Health, Tsinghua University, Beijing, China; ^5^ Department of Psychiatry and Behavioral Sciences, Emory University, Atlanta, GA, United States; ^6^ Institute for Hospital Management of Tsinghua University, Beijing, China; ^7^ Atlanta Veterans Affairs Medical Center, Decatur, GA, United States

**Keywords:** smoking, mental health professionals, burnout, health behaviors, China

## Abstract

**Objectives:**

Mental health professionals (MHPs) play an important role in treating patients with nicotine addiction. However, data on MHPs’ cigarette smoking habits are scarce. This survey aimed to collect such data and to examine the correlates of smoking among MHPs working in China.

**Methods:**

A cross-sectional survey was conducted in all 41 provincial, tertiary psychiatric hospitals, and MHPs from these hospitals were targeted. An anonymous questionnaire was designed to collect socio-demographic and occupational factors, and the Maslach Burnout Inventory-Human Service Survey was used to assess burnout. Data about smoking and other health behaviors were also collected.

**Results:**

In total, 13,614 MHPs were included in the analysis. The overall rate of current smoking was 8.6% (31.3% in males, and 1.1% in females). A substantial proportion (28.0%) of life-time smokers had abstained for more than 3 months. Those who were male (OR=37.73), older (OR=1.02), divorced or widowed (OR=1.72), working in West (OR=1.45), and Northeast China (OR=1.65), were nurses (OR=1.44), had a high income (OR=1.31), experienced burnout (OR=1.29), frequent insomnia (OR=1.39), and used alcohol (OR=2.76) were significantly more likely to be smokers, while those who had a higher level of education (OR=0.67, 0.47, and 0.43 for college, master, and doctorate degrees, respectively), and exercised regularly (OR=0.73) were significantly less likely to be smokers.

**Conclusion:**

Although lower than that of the general population in China, smoking is still relatively high among MPHs. Efforts to lower smoking rates among MHPs in China should continue and should incorporate strategies that target burnout, sleep, alcohol use, exercise and other factors associated with smoking.

## Introduction

Tobacco use is one of the most serious public health problems in the world. Six million deaths per year are associated with tobacco use worldwide, and more than 1 million of those occur in China (1, 2). In the past few decades, China has accounted for 40% of the world’s production and consumption of cigarettes, far more than any other country (2). According to the 2019 WHO report on the tobacco epidemic, 27.7% of people in China aged 15 years and older were current smokers (52.1% of males, and 2.7% of females, men/women ratio = 19.3) (3).

The role of healthcare professionals in antismoking campaigns and patients’ smoking cessation efforts is uniquely important. First, people often consciously or subconsciously view healthcare professionals as role models (4, 5). So, if the health professional smokes, then the patient might be less concerned about the dangers of nicotine use and might feel no urgency to quit. Second, healthcare providers’ own smoking behavior may affect how they approach their patients’ cigarette smoking (6), leading them to be more sympathetic to their patients’ need to smoke and tenuous in their efforts to convince the patient to give up nicotine. Multiple studies (7–10) have found high rates of smoking rates among patients with mental illness with the highest rates seen among those with bipolar disorder and schizophrenia (over 60%). Given this high prevalence and the influence health care professionals have on their patients’ smoking cessation efforts, it is important to characterize the smoking behavior of mental health professionals (MHPs), which include psychiatrists, psychiatric nurses, and psychologists.

So far, there have been several large sample surveys conducted outside of China (New Zealand, Spain, Japan, U.S., Ireland and Italy) assessing smoking behavior among doctors and nurses (11–16). The findings showed that the rates of smoking rates ranged widely, from 2%-31% for doctors and 2%–41% for nurses depending on the country studied and the criteria used. Related factors such as sex, age, profession, and health behaviors/lifestyles (such as alcohol use, skipping breakfast, general health and poor sleep) were found to be significantly associated with smoking (11, 15–17). Additionally, several studies also showed a significant association between smoking behavior and burnout among healthcare workers in Italy (18), and physicians and nurses in Saudi Arabia (19). Smoking can be seen as an option for healthcare workers to deal with feelings of exhaustion related to work problems (18). On the contrary, another study in Kazakhstan showed that smoking did not predict high burnout among doctors and nurses (20). The relationship between smoking and burnout in Chinese healthcare workers, especially in MPHs has never been explored.

Similar surveys have also been conducted in China. For example, a national survey involving 39,248 Chinese physicians in hospitals of different levels found that 20.4% overall were current smokers (38.7% in males and 1.1% in females), but the results did not include data on psychiatrists’ smoking behaviors. Regarding smoking among nurses in China, two recent studies showed the current smoking rates were from 0.9% to 1.2% among nurses in general hospitals (21, 22). One of the studies reported a smoking rate of 13.4% among psychiatric nurses in China, with a relatively small sample size of 387 (21). To date, no studies have specifically investigated cigarette smoking among MHPs in a national sample in China. This study aimed to investigate the prevalence, sociodemographic and occupational correlates of smoking among Chinese MHPs, and to explore the relationship of smoking with burnout and other health behaviors of MHPs.

## Methods

### Study Design and Participants

This cross-sectional study was part of a national survey, which was conducted in March 2019. Forty-one tertiary psychiatric hospitals from 29 provinces were selected as targets and all doctors, nurses and psychologists in these hospitals were invited to participate. Each Provincial Health Commission issued a notice to the selected hospitals, and then hospital administrators organized and facilitated healthcare professionals to participate in this survey. Each MHP completed a smartphone-based questionnaire anonymously and voluntarily throughout the process. Gansu and Tibet provinces had no provincial tertiary psychiatric hospitals and were excluded in the survey.

The research protocol was approved by the Ethics Committee of Chaohu Hospital of Anhui Medical University and an electronic consent form was obtained from each participant.

### Questionnaires

The questionnaire consisted of three parts. Clear instructions were provided to participants before each section. The first part involved socio-demographic and occupational characteristics such as age (years), sex (male/female), marital status (married, single, divorced or widowed), region (East, Central, West, or Northeast China), education (degree), profession (doctor, nurse, or psychologist), and monthly income (low: ≤5,000, medium: 5,000–10,000, or high: >10,000 RMBs). We regrouped the monthly income levels based on the most recent Chinese income standards (23). We followed the current classification of economic regions in China according to the National Bureau of Statistics (24).

In part 2, the Maslach Burnout Inventory-Human Service Survey (MBI-HSS) (25) was used to measure burnout among MHPs. The Chinese version of MBI-HSS has been validated in many studies (26–28). This is a 22-item scale scoring the following three domains of burnout: emotional exhaustion (EE), involving ninr items; depersonalization (DP), involving 5 items; and reduced personal accomplishment (PA), involving eight items. These items were scored on a 7-point scale from 0 to 6 according to frequency of symptoms. Participants with high EE (≥ 27) and/or DP (≥ 10) scores were defined as “having burnout” (27).

The third part included health-related behaviors (29), such as physical exercise, sleep, alcohol use, and cigarette smoking. In this study, we defined “regular exercise” as exercising at least 3 times per week according to the recommendations of the National Fitness Guideline (30), “frequent insomnia” as having sleep disturbances (difficulty falling asleep, difficulty maintaining sleep, or waking up early) at least 3 times per week (31), and “alcohol use” as having an alcoholic drink at least twice a month during the past year.

Questions about smoking in this study only refer to cigarette smoking, since it is by far the most common type of tobacco use in China (current cigarette/electronic-cigarette use ratio =54.6) (3). We asked about cigarette smoking status and there were three answers: never, past, and current smoking. We provided a descriptive definition for each answer: “smoking” was defined as cumulative smoking of at least 100 cigarettes (13), and “past (former or ex) smoking” was defined as a continuous cessation of smoking for more than 3 months (14). If participants answered “current smoking”, then they were asked another question, “how many cigarettes did you smoke every day in the past month?” Individuals who smoked at least 20 cigarettes daily were defined as “heavy smokers” (14). Furthermore, the duration of smoking was recorded. Finally, current smokers’ intention to quit was also investigated.

### Data Analysis

Since the numerical variables between different groups were not normally distributed (Kolmogorov-Smirnov test, all *p* < 0.05), we compared the differences between groups using the Kruskal-Wallis H test (3 groups) or Mann-Whitney U test (2 groups) for continuous variables and chi-square test for categorical variables. Then, we compared the prevalence of burnout, regular exercise, frequent insomnia, and alcohol use between smoking (current) and non-smoking groups using logistic regression analyses to control for confounding factors. We applied Bonferroni correction to the comparisons of these rates to adjust for multiple tests. Finally, binary logistic regression analysis was used to determine the independent factors of smoking within the whole sample. As sex is considered to be an important factor associated with smoking, we conducted a further logical regression analysis of male participants. Since there were only 110 smokers among the female participants, the same analysis was not performed. We conducted statistical analyses using SPSS version 23.0 with the significance level at *p* value of 0.05 (2- tailed).

## Results

### Participant Characteristics

A total of 21,858 MHPs, including 6,986 doctors (73.3% were psychiatrists), 13,867 nurses (78.8% were registered psychiatric nurses), and 1,005 psychologists were invited to participate in this survey. 14,666 responded (response rate = 67.1%) and 13,614 (62.3%) (4,345 doctors, 9,112 nurses and 257 psychologists) completed the questionnaire with no logical errors and were included in the statistical analysis. The mean age of the total sample was 35.8 ± 8.8 years (range, 19–90 years). Two thirds (66.9%) were nurses and three quarters were female (75.1%) and married (75.3%). Significant group difference was found in several socio-demographic and occupational variables, such as age, education, marital status, region, profession, and monthly income (all *p* < 0.05) (**Table 1**).

**Table 1:** Sociodemographic characteristics of the participating mental health professionals.

	Allparticipants	Current smokers	Pastsmokers	Neversmokers	Statistics	
	*n*=13,614	*n*=1,170	*n*=454	*n*=11,990	H or χ^2^	*p* value
Age (years) (Mean ± SD)	35.8 ± 8.8	37.7 ± 9.6	38.4 ± 10.2	35.5 ± 8.6	79.24^a^	<0.001
Sex (%)					4058.39^b^	<0.001
Male	3,392 (24.9)	1,060 (90.6)	386 (85.0)	1,946 (16.2)		
Female	10,222 (75.1)	110 (9.4)	68 (15.0)	10,044 (83.8)		
Education level (%)					65.71^b^	<0.001
Associate degree or less	3,199 (23.5)	365 (31.2)	142 (31.3)	2,692 (22.5)		
College degree^c^	8,888 (65.3)	699 (59.7)	276 (60.8)	7913 (66.0)		
Master’s degree	1285 (9.4)	86 (7.4)	30 (6.6)	1169 (9.7)		
Doctorate degree	242 (1.8)	20 (1.7)	6 (1.3)	216 (1.8)		
Marital status (%)					25.21^b^	<0.001
Married	10,258 (75.3)	895 (76.5)	370 (81.5)	8,993 (75.0)		
Single	2,868 (21.1)	219 (18.7)	63 (13.9)	2,586 (21.6)		
Divorced or widowed	488 (3.6)	56 (4.8)	21 (4.6)	411 (3.4)		
Region (%)					49.63^b^	<0.001
East China	5,148 (37.8)	364 (31.1)	158 (34.8)	4,626 (38.6)		
Central China	2,523 (18.5)	219 (18.7)	77 (17.0)	2,227 (18.6)		
West China	3,537 (26.0)	317 (27.1)	113 (24.9)	3,107 (25.9)		
Northeast China	2,406 (17.7)	270 (23.1)	106 (23.3)	2,030 (16.9)		
Profession (%)					104.24^b^	<0.001
Doctors	4,245 (31.2)	501 (42.8)	181 (39.9)	3,563 (29.7)		
Nurses	9,112 (66.9)	657 (56.2)	267 (58.8)	8,188 (68.3)		
Psychologists	257 (1.9)	12 (1.0)	6 (1.3)	239 (2.0)		
Income (%)^d^					23.06^b^	<0.001
Low	5,269 (38.7)	425 (36.3)	153 (33.7)	4,691 (39.1)		
Middle	6,832 (50.2)	573 (49.0)	243 (53.5)	6,016 (50.2)		
High	1,513 (11.1)	172 (14.7)	58 (12.8)	1,283 (10.7)		

^a^Kruskal-Wallis H test; ^b^Chi-square test; ^c^Including medical school in China; ^d^Monthly income, low: ≤5,000, medium: 5,000–10,000, or high: >10,000 RMBs, one dollar is approximately equal to 6.95 RMBs at the time of study.

### The Prevalence of Current and Past Smoking

The overall prevalence of current and past smoking was 8.6% (31.3% in males and 1.1% in females, same hereafter) and 3.3% (11.4% and 0.7%) in the entire sample, respectively. **Table 2** showed the prevalence of current and past smoking by gender, profession, and age. Using the sum of current and past smokers as life-smokers, we calculated the percentages of past smokers among life-time smokers. It was 28.0% in the whole sample, 26.5% among doctors, 28.9% among nurses, and 33.3% among psychologists. The highest prevalence of current and past smoking was found in the age group, ≥ 50 years (14.0% and 6.3%). The prevalence of heavy smoking was 27.5% in current smokers (28.9% and 14.5%). The mean duration of smoking was 13.9 ± 8.8 years in current smokers and 9.3 ± 7.7 years in past smokers. In current smokers, 20.8% (244/1170) reported an intention to quit within 1 year, and 29.7% (347/1170) reported no intention to quit.

**Table 2:** The prevalence of current and past cigarette smoking by gender, professions and age.

Profession	Doctors	Nurses	Psychologists	Total	χ^2^	*p* value
Male	*n*=1,786	*n*=1,566	*n*=40	*n*=3,392		
Current smoker^a^	490 (27.4)	559 (35.7)	11 (27.5)	1060 (31.3)	26.77	<0.001
Past smoker^a^	176 (9.9)	206 (13.2)	4 (10.0)	386 (11.4)	8.98^b^	0.01
Female	*n*=2,459	*n*=7,546	*n*=217	*n*=10,222		
Current smoker^a^	11 (0.4)	98 (1.3)	1 (0.5)	110 (1.1)	14.64^b^	0.001
Past smoker^a^	5 (0.2)	61 (0.8)	2 (0.9)	68 (0.7)	12.75^b^	0.002
**Age**	**19–29, years**	**30–39, years**	**40-49, years**	**≥50, years**	**χ^2^**	***p* value**
Male	*n*=758	*n*=1,503	*n*=650	*n*=481		
Current smoker^a^	232 (30.6)	432 (28.7)	219 (33.7)	177 (36.8)	13.24	0.004
Past smoker^a^	73 (9.6)	152 (10.1)	83 (12.8)	78 (16.2)	17.09	0.001
Female	*n*=2,810	*n*=4,569	*n*=1,961	*n*=882		
Current smoker^a^	30 (1.1)	47 (1.0)	19 (1.0)	14 (1.6)	2.48	0.48
Past smoker^a^	15 (0.5)	36 (0.8)	9 (0.5)	8 (0.9)	3.82	0.28

^a^Data are presented as number of professionals (percentage); ^b^Fisher’s Exact Test.

### Burnout and Other Health Behaviors

**Figure 1:**
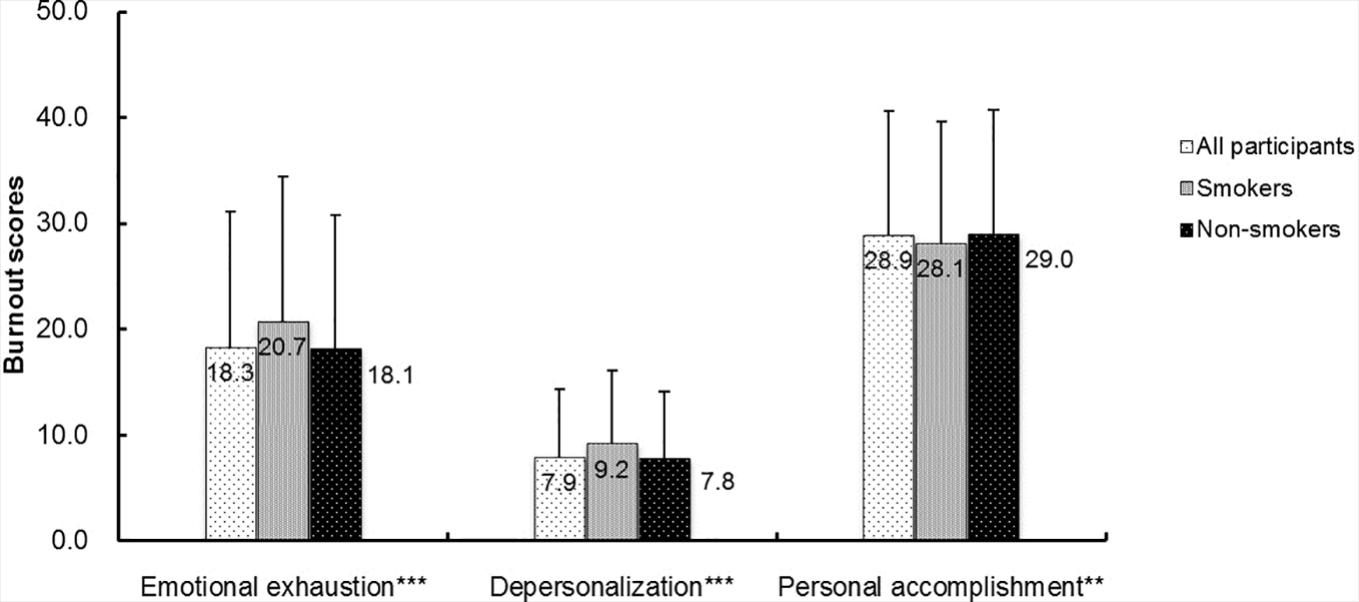
The comparisons of burnout scores between smokers and non-smokers among the participating mental health professionals. **p < 0.01; ***p < 0.001 by Mann-Whitney U-test. The *p* values were comparison between smokers and non-smokers.

We found significant differences in scores on EE, DP, and PA between smokers and non-smokers (all *p* < 0.01) (**Figure 1**). The rates of burnout, regular exercise, frequent insomnia, and alcohol use in the whole sample were 38.1%, 7.2%, 26.9%, and 10.2%, respectively. Compared with non-smokers, smokers had higher rates of burnout (45.6% *vs* 37.4%, χ^2 =^ 31.05, *p* < 0.001), frequent insomnia (36.8% *vs* 25.9%, χ^2^ = 63.73, *p* < 0.001), and alcohol use (41.9% vs 7.2%, χ^2^ = 1397.34, *p* < 0.001). After Bonferroni correction (α=0.05/4 = 0.0125), all differences in these rates remained significant (all *p* < 0.0125). After controlling for the socio-demographic and occupational confounders, including age, education, marital status, region, profession, and monthly income, significant associations were found between smoking and burnout (OR = 1.45, 95%CI: 1.27–1.67, *p* < 0.001), frequent insomnia (OR = 1.56, 95%CI: 1.35-1.81, *p* < 0.001), regular exercise (OR = 0.72, 95%CI: 0.56–0.91, *p* = 0.007), and alcohol use (OR = 2.79, 95%CI: 2.39–3.26, *p* < 0.001).

### Factors Associated With Smoking

We conducted a multiple logistic regression analysis with current smoking as the dependent variable and the other related variables as covariates. The reference category for each categorical covariate is shown in **Table 3**. The results showed that an older age, male sex, being divorced or widowed, working in West and Northeast China, being nurses, having a high income, burnout, frequent insomnia, and alcohol use were all significantly associated with a higher risk of smoking, while higher levels of education and exercising regularly were associated with a lower risk of smoking (all *p* < 0.05). The regression analysis of male participants showed that except income levels (*p* > 0.05), other factors were still significantly associated with smoking, which was consistent with the results in the whole sample (all *p* < 0.05).

**Table 3:** Multiple logistic regression of factors associating with smoking in mental health professionals.

Variables	Coefficient	Std. Error	Wald χ2	*p* value	OR (95% CI)
Age (years)	0.02	0.01	9.62	0.002	1.02 (1.01-1.03)
Male (ref. female)	3.63	0.11	1099.99	<0.001	37.73 (30.45-46.76)
Education level (ref. associate degree or less)					
College degree	-0.41	0.10	18.28	<0.001	0.67 (0.55-0.80)
Master’s degree	-0.76	0.17	20.41	<0.001	0.47 (0.34-0.65)
Doctorate degree	-0.85	0.29	8.83	0.003	0.43 (0.24-0.75)
Marital status (ref. married)					
Single	0.01	0.11	0.11	0.91	1.01 (0.82-1.25)
Divorced or widowed	0.54	0.19	8.33	0.004	1.72 (1.19-2.48)
Region (ref. East China)					
Central China	0.10	0.11	0.95	0.33	1.11 (0.90-1.37)
West China	0.37	0.10	13.75	<0.001	1.45 (1.19-1.77)
Northeast China	0.50	0.12	18.80	<0.001	1.65 (1.32-2.07)
Profession (ref. doctors)					
Nurses	0.37	0.10	13.68	<0.001	1.44 (1.19-1.75)
Psychologists	0.22	0.35	0.39	0.53	1.24 (0.63-2.44)
Income (ref. low income)					
Middle	0.06	0.09	0.43	0.51	1.06 (0.89-1.26)
High	0.27	0.13	3.96	0.047	1.31 (1.00-1.70)
Burnout (ref. no)	0.26	0.08	11.04	0.001	1.29 (1.11-1.50)
Regular exercise (ref. no)	-0.31	0.13	5.90	0.02	0.74 (0.57-0.94)
Frequent insomnia (ref. no)	0.33	0.08	16.20	<0.001	1.39 (1.18-1.63)
Alcohol use (ref. no)	1.02	0.08	160.89	<0.001	2.76 (2.36-3.23)
Constant	-5.63	0.29	366.08	<0.001	0.004

R^2^ = 0.436; CI, confidence interval.

## Discussion

This was the first national survey to investigate the prevalence of current and past cigarette smoking and their correlates among Chinese MHPs. We examined the demographic correlates as well as the association between smoking and burnout, and other health-related behaviors such as exercise, sleep and alcohol use. A few strengths of this study should be mentioned. First, our sample size is by far the largest involving MPHs and the online survey format and anonymity likely encouraged participants’ honest responses. We targeted 41 top-tier psychiatric hospitals from 29 provinces and the participation rate at the hospital level was 100%. MHPs in these hospitals accounted for approximatively one-fifth of the total registered doctors and nurses working within psychiatric hospitals in China (32). Second, we collected data on both current and past smoking behavior and we used clear descriptive definitions for all items. Third, we collected data on burnout and health-related behaviors so we could examine their associations with smoking.

We found the rate of current smoking was 8.6% in the whole sample (31.3% in males, and 1.1% in females) and the rate of past smoking (smoking cessation ≥3 months) was 3.3% (11.4% in males and 0.7% in females). We found a striking sex difference with males being 37.7 times more likely to be a smoker than females. We also found cigarette use was significantly associated with demographic factors such as older age, being male, divorced or widowed, and working in West and Northeast regions. Importantly, we also found cigarette smoking was significantly associated with burnout, insomnia and alcohol use.

In this study, the prevalence of cigarette smoking among MHPs was lower than that in the general population of China (31.3% vs 51.4% in males; 1.1% vs 2.7% in females) (3). On the other hand, we found relatively high rates of past smoking (i.e. successfully quit smoking for at least 3 months) in our sample across different professions and age groups: it was 13.2% in male nurses, 0.8% in female nurses, and 16.2% in male participants older than 50 years. The ratio of male past smokers to current smokers in our study (11.4% to 31.3%) is higher than that reported in previous study (8.4% to 54.0%) (33). Furthermore, compared with the general population of China, MHPs have a lower proportion of heavy smokers (27.5% vs 51.3%), and a higher proportion of smokers with an intention to quit (20.8% vs 7.9%) (34). These findings suggest that healthcare professionals’ overall awareness of smoking risks and access to smoking cessation treatments (35) may be better than that of the general population, or there may be greater pressure among MHPs to quit because of their role as healthcare workers.

The striking sex difference in smoking is consistent with most studies from China (21, 36–38), and reflects a cultural stereotype around smoking and alcohol use (39, 40). Traditionally, smoking and alcohol use have been considered masculine behaviors and women who engage in them are often strongly discouraged or stigmatized for doing so. However, recent data show that there has been a slight increase in cigarette use among women (from 2.5% in 2010 to 2.7% in 2015) (41). Additionally, smoking is considered a symbol of showing-off and independence (42). Our data revealed that, although still very low compared to their male counterparts, the rate of smoking in female nurses was more than three times the rate in female doctors (1.3% vs 0.4%), and the ratio between female and male nurses (1:27.5) was higher than that in doctors (1:68.5).

The rates of smoking in male and female doctors in our sample were 27.4% and 0.4%, respectively, and they were both lower than those seen among doctors working in general tertiary hospitals in China (35.9% in males, and 1.1% in females) (36). There are two possible explanations for this discrepancy. First, about 10 years ago, China initiated a series of public campaigns against smoking in public places, including restaurants, schools and hospitals (43). The Jiang et al. (36) study was conducted before the launch of a major public antismoking campaign, so the rates were expected to be higher. Second, the samples in the Jiang study included surgeons (41.4% and 2.7%) and dentists (42.2% and 0.8%), two specialties known for their high rates of smoking. The rate of smoking in male doctors in this study is significantly higher than that in New Zealand (2%) (11) and Spain (8.9%) (15), and it is similar to that in Japan (27.1%) (16) and Italy (29%) (14). It is worth noting that the samples from these countries were not limited to health professionals in psychiatric hospitals. Of note, the smoking rate among male nurses in our sample was particularly high (35.7%). This is higher than that seen among male nurses working in Ireland (25%) (12), but lower than the rate reported among 283 Italian professional nurses (40%) (14). This elevated rate may be due to the high levels of stress experienced by male psychiatric nurses in China. Our previous study showed that within a 12-month period, one-third of psychiatric nurses in China experienced patient-initiated violence (44).

Furthermore, we found that an older age, a lower level of education and being divorced or widowed were associated with smoking among MHPs. Young health professionals in China are provided universal education on the harms of smoking and tend to have positive attitudes towards smoking bans (45), both of which may account for their lower smoking rates. Perhaps for the same reasons, those with higher levels of education have been more successful in quitting smoking than those with lower levels of education (46).Previous studies found that being divorced or separated was more likely to be associated with lower life satisfaction and predicted a higher risk of smoking than being married (29, 47). In addition, we found significant regional differences in smoking, with higher rates in West and Northeast China than those in East China. This may reflect the cultural differences that exist between different regions of the country (48).

One new finding in our study is the significant association between smoking and burnout. We found that a MHP meeting criteria for burnout had a 1.3 greater odds of being a smoker than a non-smoker. This finding is consistent with the results of previous studies conducted in other countries (18, 19). Burnout of health professionals often coexists with low job satisfaction (26), and leads to negative emotions (28) and negative lifestyle choices, including smoking (18). Smoking is also commonly used as a coping method for anxiety symptoms and negative affect (49, 50).

The substantial link between alcohol and tobacco use has been repeatedly confirmed (51, 52). Our results showed that drinkers are more likely (OR=2.76) to smoke than non-drinkers. Especially in some areas in China, such as the Northeast, the co-use of tobacco and alcohol increases the smoking rate of the local people, in that they often only smoke when using alcohol (48). This, along with our findings associating smoking with other health-related behaviors and symptoms (e.g. drinking, lack of regular exercise, insomnia) suggests that strategies targeting smoking cessation must incorporate additional health-promoting interventions. For example, a previous study showed that people with poor sleep may self-medicate and try to improve their mood using nicotine (53). Another study found that among heavy smokers who also drank alcohol, reducing their alcohol use helped them quit their daily smoking habit (54). An earlier study showed vigorous exercise facilitates short- and longer-term smoking cessation in women when combined with a cognitive-behavioral smoking cessation program (55). As these behaviors are all inter-related with each other, targeting only one aspect is unlikely to succeed on its own.

Several limitations in our study should also be noted. First, we only surveyed cigarette smoking and other types of tobacco use (although relatively rare) were not included in our questionnaire. Second, we collected data based on self-report and we did not use validated instruments (such as the Fagerström Test for Nicotine Dependence) or objective measures for smoking (such as cotinine levels). In addition, possible biases may exist due to the nature of self-report measurements, and due to the fact that some MHPs declined to participate or did not complete the survey. Third, as is the case in most cross-sectional surveys, our data were not able to address the direction of causality. Finally, the participants in our study were MHPs working in top-tier psychiatric hospitals and our findings may not generalize to other populations of MHPs in China.

## Conclusions

In conclusion, based on this survey of a large, national sample of MHPs from 41 tertiary psychiatric hospitals across China, we found the overall rate of cigarette smoking was 8.6% (31.3% in males, and 1.1% in females), which was lower than that seen in the general population. Additionally, cigarette smoking among MHPs was associated with a number of socio-demographic factors, burnout levels and health-related behaviors, including alcohol use, insomnia, and lack of regular exercise. Longitudinal studies using validated questionnaires or objective measures for smoking are necessary to further clarify the relationship of cigarette smoking with these factors in Chinese MHPs. Given their work as healthcare professionals who often address nicotine addiction and the dangers of smoking with their own patients, efforts to lower smoking rates among MHPs in China should continue and should incorporate strategies that target burnout, sleep, alcohol use, exercise and other factors associated with smoking cigarettes.

## Data Availability Statement 

The data used for this study are available from the corresponding authors on reasonable request.

## Ethics Statement 

The studies involving human participants were reviewed and approved by the Ethics Committee of Chaohu Hospital of Anhui Medical University. The patients/participants provided their written informed consent to participate in this study.

## Author Contributions

FJ, HL, YL, and Y-LT conceived and planned the survey. LX, YZ, and KZ contributed to the implementation and acquisition of data for this study. LX and FJ did all data analyses and drafted the manuscript. Y-LT and JR gave many useful suggestions and amended the manuscript. All authors contributed to the article and approved the submitted version.

## Funding

This research was funded by the Beijing Medical and Health Foundation (Grant No. MH180924), and the National Clinical Key Specialty Project Foundation (CN).

## Conflict of Interest

The authors declare that the research was conducted in the absence of any commercial or financial relationships that could be construed as a potential conflict of interest.

## References

[1] Cahn Z Drope J Hamill S Islami F Liber A Nargis N The Tobacco Atlas: Sixth Edition. Atlanta: American Cancer Society (2018).

[2] National Health Commission of China Report on the health hazards of smoking in China. Beijing: People’s Health Publishing House (2012).

[3] World Health Organization (WHO) WHO report on the global tobacco epidemic. Geneva: World Health Organization (2019). https://www.who.int/tobacco/surveillance/policy/country_profile/chn.pdf?ua=1 [Accessed April 4, 2020].

[4] Abdullah AS Stillman FA Yang L Luo H Zhang Z Samet JM Tobacco use and smoking cessation practices among physicians in developing countries: a literature review (1987-2010). Int J Environ Res Public Health (2013) 11:429–55. 10.3390/ijerph110100429 PMC3924453 24380976

[5] Pipe A Sorensen M Reid R Physician smoking status, attitudes toward smoking, and cessation advice to patients: an international survey. Patient Educ Couns (2009) 74:118–23. 10.1016/j.pec.2008.07.042 18774670

[6] Duaso MJ McDermott MS Mujika A Purssell E While A Do doctors’ smoking habits influence their smoking cessation practices? A systematic review and meta-analysis. Addiction (2014) 109:1811–23. 10.1111/add.12680 25041084

[7] Lasser K Boyd JW Woolhandler S Himmelstein DU McCormick D Bor DH Smoking and mental illness: A population-based prevalence study. JAMA (2000) 284:2606–10. 10.1001/jama.284.20.2606 11086367

[8] Gonzalez-Pinto A Gutierrez M Ezcurra J Aizpuru F Mosquera F Lopez P Tobacco smoking and bipolar disorder. J Clin Psychiatry (1998) 59:225–8. 10.4088/jcp.v59n0503 9632031

[9] de Leon J Diaz FJ A meta-analysis of worldwide studies demonstrates an association between schizophrenia and tobacco smoking behaviors. Schizophr Res (2005) 76:135–57. 10.1016/j.schres.2005.02.010 15949648

[10] Tang YL George TP Mao PX Cai ZJ Chen Q Cigarette smoking in Chinese male inpatients with schizophrenia: a cross-sectional analysis. J Psychiatr Res (2007) 41:43–8. 10.1016/j.jpsychires.2005.10.009 16360170

[11] Edwards R Tu D Stanley J Martin G Gifford H Newcombe R Smoking prevalence among doctors and nurses-2013 New Zealand census data. N Z Med J (2018) 131:48–57. 29518799

[12] O’Donovan G Smoking prevalence among qualified nurses in the Republic of Ireland and their role in smoking cessation. Int Nurs Rev (2009) 56:230–6. 10.1111/j.1466-7657.2008.00700.x 19646173

[13] Tong EK Strouse R Hall J Kovac M Schroeder SA National survey of U.S. health professionals’ smoking prevalence, cessation practices, and beliefs. Nicotine Tob Res (2010) 12:724–33. 10.1093/ntr/ntq071 PMC6281036 20507899

[14] Zanetti F Gambi A Bergamaschi A Gentilini F De Luca G Monti C Smoking habits, exposure to passive smoking and attitudes to a non-smoking policy among hospital staff. Public Health (1998) 112:57–62. 10.1038/sj.ph.1900419 9490891

[15] Jimenez-Ruiz CA Riesco Miranda JA Ramos Pinedo A de Higes Martinez E Marquez FL Palomo Cobos L Prevalence of and Attitudes towards Smoking among Spanish Health Professionals. Respiration (2015) 90:474–80. 10.1159/000441306 26484660

[16] Ohida T Sakurai H Mochizuki Y Kamal AM Takemura S Minowa M Smoking prevalence and attitudes toward smoking among Japanese physicians. JAMA (2001) 285:2643–8. 10.1001/jama.285.20.2643 11368741

[17] Fujita Y Maki K Associations of smoking behavior with lifestyle and mental health among Japanese dental students. BMC Med Educ (2018) 18:264. 10.1186/s12909-018-1365-1 30445940 PMC6472720

[18] Petrelli F Scuri S Tanzi E Nguyen C Grappasonni I Public health and burnout: a survey on lifestyle changes among workers in the healthcare sector. Acta BioMed (2018) 90:24–30. 10.23750/abm.v90i1.7626 30889151 PMC6502147

[19] Alqahtani AM Awadalla NJ Alsaleem SA Alsamghan AS Alsaleem MA Burnout Syndrome among Emergency Physicians and Nurses in Abha and Khamis Mushait Cities, Aseer Region, Southwestern Saudi Arabia. Sci World J (2019) 2019:4515972. 10.1155/2019/4515972 PMC6398028 30906233

[20] Vinnikov D Dushpanova A Kodasbaev A Romanova Z Almukhanova A Tulekov Z Occupational burnout and lifestyle in Kazakhstan cardiologists. Arch Public Health (2019) 77:13. 10.1186/s13690-019-0345-1 31007909 PMC6457002

[21] An FR Xiang YT Yu L Ding YM Ungvari GS Chan SW Prevalence of nurses’ smoking habits in psychiatric and general hospitals in China. Arch Psychiatr Nurs (2014) 28:119–22. 10.1016/j.apnu.2013.11.008 24673786

[22] Zhou W Chao Y Zhang H Ye D Ding Y Ke H Analysis of smoking behavior and its influencing factors among medical staff in a tertiary hospital in Beijing (in Chinese). Chin J Prev Contr Chron Dis (2019) 27:687–9. 10.16386/j.cjpccd.issn.1004-6194.2019.09.012

[23] National Bureau of Statistics of China Interpretation of the bulletin of the national survey on the use of time in 2018. Beijing: National Bureau of Statistics of China (2019). http://www.gov.cn/xinwen/2019-01/25/content_5361066.htm [Accessed April 4, 2020].

[24] National Bureau of Statistics of China Division method of East, West, Central and Northeast China. Beijing: National Health Commission of China (2011). http://www.stats.gov.cn/ztjc/zthd/sjtjr/dejtjkfr/tjkp/201106/t20110613_71947.htm [Accessed June 1, 2020].

[25] Maslach C Jackson SE Leiter MP Maslach burnout inventory manual (3rd ed.). Palo Alto, CA: Consulting Psychologists Press (1996).

[26] Li H Zuo M Gelb AW Zhang B Zhao X Yao D Chinese Anesthesiologists Have High Burnout and Low Job Satisfaction: A Cross-Sectional Survey. Anesth Analg (2018) 126:1004–12. 10.1213/ane.0000000000002776 29309320

[27] Ma S Huang Y Yang Y Ma Y Zhou T Zhao H Prevalence of Burnout and Career Satisfaction Among Oncologists in China: A National Survey. Oncologist (2019) 24:e480–e9. 10.1634/theoncologist.2018-0249 PMC6656451 30568022

[28] Zheng H Shao H Zhou Y Burnout Among Chinese Adult Reconstructive Surgeons: Incidence, Risk Factors, and Relationship With Intraoperative Irritability. J Arthroplasty (2018) 33:1253–7. 10.1016/j.arth.2017.10.049 29239771

[29] Molloy GJ Stamatakis E Randall G Hamer M Marital status, gender and cardiovascular mortality: behavioural, psychological distress and metabolic explanations. Soc Sci Med (2009) 69:223–8. 10.1016/j.socscimed.2009.05.010 PMC2852675 19501442

[30] General Administration of Sport of China National Fitness Guideline. Beijing: China Sports Daily (2017). http://www.sport.gov.cn/n316/n337/c819036/content.html [Accessed April 4, 2020].

[31] Li SX Lam SP Zhang J Yu MW Chan JW Chan CS Sleep Disturbances and Suicide Risk in an 8-Year Longitudinal Study of Schizophrenia-Spectrum Disorders. Sleep (2016) 39:1275–82. 10.5665/sleep.5852 PMC4863217 27091530

[32] National Health Commission of China Chinese Health Statistical Yearbook 2019. Beijing: Peking Union Medical College Press (2019).

[33] Liu S Zhang M Yang L Li Y Wang L Huang Z Prevalence and patterns of tobacco smoking among Chinese adult men and women: findings of the 2010 national smoking survey. J Epidemiol Community Health (2017) 71:154–61. 10.1136/jech-2016-207805 PMC5284482 27660401

[34] Qian J Cai M Gao J Tang S Xu L Critchley JA Trends in smoking and quitting in China from 1993 to 2003: National Health Service Survey data. Bull World Health Organ (2010) 88:769–76. 10.2471/blt.09.064709 PMC2947036 20931062

[35] Sheng LX Tang YL Jiang ZN Yao CH Gao JY Xu GZ Sustained-release bupropion for smoking cessation in a Chinese sample: a double-blind, placebo-controlled, randomized trial. Nicotine Tob Res (2013) 15:320–5. 10.1093/ntr/nts124 22614545

[36] Jiang Y Li X Wu X Li Q Yang Y Nan Y Smoking behavior of Chinese physician (in Chinese). Chin J Prev Contr Chron Dis (2009) 17:224–7. 10.16386/j.cjpccd.issn.1004-6194.2009.03.002

[37] Smith DR Zhao I Wang L Tobacco smoking among doctors in mainland China: a study from Shandong province and review of the literature. Tob Induc Dis (2012) 10:14. 10.1186/1617-9625-10-14 23006640 PMC3519549

[38] Li Q Hsia J Yang G Prevalence of smoking in China in 2010. N Engl J Med (2011) 364:2469–70. 10.1056/NEJMc1102459 21696322

[39] Li S Meng L Chiolero A Ma C Xi B Trends in smoking prevalence and attributable mortality in China, 1991-2011. Prev Med (2016) 93:82–7. 10.1016/j.ypmed.2016.09.027 PMC5124560 27677441

[40] Millwood IY Li L Smith M Guo Y Yang L Bian Z Alcohol consumption in 0.5 million people from 10 diverse regions of China: prevalence, patterns and socio-demographic and health-related correlates. Int J Epidemiol (2013) 42:816–27. 10.1093/ije/dyt078 PMC3733702 23918852

[41] Chinese Centre for Disease Control and Prevention 2015 China Adult Tobacco Survey. Beijing: Chinese Centre for Disease Control and Prevention (2016). http://www.tcrc.org.cn/html/zy/cbw/jc/3259.html [Accessed April 4, 2020].

[42] Baheiraei A Mirghafourvand M Mohammadi E Majdzadeh R Experiences of Cigarette Smoking among Iranian Educated Women: A Qualitative Study. Int J Prev Med (2016) 7:93. 10.4103/2008-7802.186585 27563429 PMC4977981

[43] National Health Commission of China Regulations on Health Administration in Public Places. Beijing: National Health Commission of China (2011). http://www.gov.cn/flfg/2011-03/22/content_1829432.htm [Accessed April 4, 2020].

[44] Jiang F Zhou H Rakofsky J Hu L Liu T Wu S Intention to leave and associated factors among psychiatric nurses in China: A nationwide cross-sectional study. Int J Nurs Stud (2019) 94:159–65. 10.1016/j.ijnurstu.2019.03.013 30978616

[45] Yang T Yu L Bottorff JL Wu D Jiang S Peng S Global Health Professions Student Survey (GHPSS) in Tobacco Control in China. Am J Health Behav (2015) 39:732–41. 10.5993/ajhb.39.5.14 26248182

[46] Wu L He Y Jiang B Zuo F Liu Q Zhang L Relationship between education levels and booster counselling sessions on smoking cessation among Chinese smokers. BMJ Open (2015) 5:e007885. 10.1136/bmjopen-2015-007885 PMC4538246 26246076

[47] Bourassa KJ Ruiz JM Sbarra DA Smoking and Physical Activity Explain the Increased Mortality Risk Following Marital Separation and Divorce: Evidence From the English Longitudinal Study of Ageing. Ann Behav Med (2019) 53:255–66. 10.1093/abm/kay038 PMC6374715 29796660

[48] Li R Wang D Chen J Chai J Tang M Regional differences in smoking, drinking, and physical activities of Chinese residents. Asia Pac J Public Health (2015) 27:NP230–9. 10.1177/1010539512437604 22357550

[49] Buckner JD Zvolensky MJ Jeffries ER Schmidt NB Robust impact of social anxiety in relation to coping motives and expectancies, barriers to quitting, and cessation-related problems. Exp Clin Psychopharmacol (2014) 22:341–7. 10.1037/a0037206 PMC4116795 24978348

[50] Watson NL VanderVeen JW Cohen LM DeMarree KG Morrell HE Examining the interrelationships between social anxiety, smoking to cope, and cigarette craving. Addict Behav (2012) 37:986–9. 10.1016/j.addbeh.2012.03.025 22507303

[51] Beard E West R Michie S Brown J Association between smoking and alcohol-related behaviours: a time-series analysis of population trends in England. Addiction (2017) 112:1832–41. 10.1111/add.13887 PMC5600127 28556467

[52] Falk DE Yi HY Hiller-Sturmhofel S An epidemiologic analysis of co-occurring alcohol and tobacco use and disorders: findings from the National Epidemiologic Survey on Alcohol and Related Conditions. Alcohol Res Health (2006) 29:162–71. PMC6527037 17373404

[53] Bellatorre A Choi K Lewin D Haynie D Simons-Morton B Relationships Between Smoking and Sleep Problems in Black and White Adolescents. Sleep (2017) 40, zsw031. 10.1093/sleep/zsw031 PMC6084753 28364464

[54] Dermody SS Hendershot CS Andrade AK Novalen M Tyndale RF Changes in Nicotine Metabolite Ratio Among Daily Smokers Receiving Treatment for Alcohol Use Disorder. Nicotine Tob Res (2020) 22:256–63. 10.1093/ntr/nty265 30561731

[55] Marcus BH Albrecht AE King TK Parisi AF Pinto BM Roberts M The efficacy of exercise as an aid for smoking cessation in women: a randomized controlled trial. Arch Intern Med (1999) 159:1229–34. 10.1001/archinte.159.11.1229 10371231

